# Genome-wide DNA methylation profiles changes associated with constant heat stress in pigs as measured by bisulfite sequencing

**DOI:** 10.1038/srep27507

**Published:** 2016-06-06

**Authors:** Yue Hao, Yanjun Cui, Xianhong Gu

**Affiliations:** 1 State Key Laboratory of Animal Nutrition, Institute of Animal Sciences, Chinese Academy of Agricultural Sciences, Beijing, People’s Republic of China

## Abstract

Heat stress affects muscle development and meat quality in food animals; however, little is known regarding its regulatory mechanisms at the epigenetic level, such as via DNA methylation. In this study, we aimed to compare the DNA methylation profiles between control and heat-stressed pigs to identify candidate genes for skeletal muscle development and meat quality. Whole-genome bisulfite sequencing was used to investigate the genome-wide DNA methylation patterns in the *longissimus dorsi* muscles of the pigs. Both groups showed similar proportions of methylation at CpG sites but exhibited different proportions at non-CpG sites. A total of 57,147 differentially methylated regions were identified between the two groups, which corresponded to 1,422 differentially methylated genes. Gene ontogeny and KEGG pathway analyses indicated that these were mainly involved in energy and lipid metabolism, cellular defense and stress responses, and calcium signaling pathways. This study revealed the global DNA methylation pattern of pig muscle between normal and heat stress conditions. The result of this study might contribute to a better understanding of epigenetic regulation in pig muscle development and meat quality.

As a result of global warming, heat stress has become a factor negatively affecting animal performance and health and may jeopardize the development and economy of animal husbandry^1^,^2^. Exposure to high ambient temperatures has been reported to be detrimental to pig production and meat quality^3^. Moreover, heat stress affects protein anabolism and catabolism as well as satellite cell proliferation, all of which play important roles in determining muscle growth and development^4^. In addition, heat stress activates signaling pathways associated with the regulation of skeletal muscle growth and development^5^,^6^.

Because the influence of stress on the success of the pig industry has increased, scientists have studied the specific effects of heat stress on pigs, particularly meat quality^7^,^8^,^9^,^10^. Heat stress is thought to affect gene transcription; appropriate regulation of gene transcription is crucial for maintenance of cell identity and physiological function. In preliminary studies, we identified some differentially expressed genes that were closely related to skeletal development and meat quality parameters between constant heat stress and normal temperature in pigs (Hao *et al.* 2015, unpublished data). However, until now, no systematic studies have evaluated the underlying molecular mechanisms mediating the development of skeletal muscle under heat stress.

DNA methylation is a well-studied epigenetic regulatory mechanism that plays a key role in the regulation of gene expression^11^. Recently, multiple studies have been conducted to identify genome-wide methylation profiles of economically important animals^12^,^13^,^14^,^15^. In particular, DNA methylation has been shown to affect the expression of many genes that are critical to skeletal muscle development^16^. Furthermore, a recent study by Li *et al.*^17^ described a genome-wide DNA methylation map and gene expression map for pig muscle, suggesting that DNA methylation status may be an epigenetic mechanism responsible for muscle growth in pigs^17^. However, to date, few studies have reported the DNA methylation patterns of skeletal muscle associated with heat stress in pigs.

The objectives of the present study were to profile genome-wide DNA methylation patterns in pig muscle and to identify methylated genes that were involved in pig muscle growth during conditions of constant heat stress. In parallel, we used high-throughput, deep-sequencing technologies to analyze heat-stress transcriptome profiles and performed an association analysis between DNA methylation levels and differentially expressed genes of the *Longissimus dorsi* muscle tissues.

## Results

### Global mapping of DNA methylation

In the present study, eight *longissimus dorsi* muscle tissues were used to generate one pooled DNA sample for each group of control (CN) and heat exposed (HE) animals. A total of 85.06 G and 78.18 G raw bases were generated for the two groups, respectively. After data filtering, 246,647,114 and 250,285,737 clean reads were generated for the two groups, respectively. The mapping reads of CN and HE covered 61.69% and 65.81% of the pig genome, respectively (Table 1).

### DNA methylation patterns

**Figure 1:**
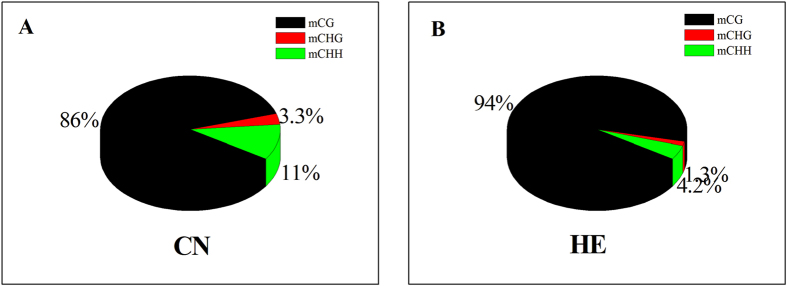
Comparison of DNA methylation patterns in the two groups.

In each group, approximately 4% of all genomic C sites were methylated. Methylation in pig was found to exist in three sequence contexts: CG, CHG (where H is A, C, or T), and CHH. We observed overall genome-wide levels of 67.62% CG, 0.59% CHG, and 0.58% CHH methylation in the CN group and 67.54% CG, 0.21% CHG, and 0.20% CHH methylation in the HE group (Table 2). Compared with the CN group, the rate of methylated CG^18^ in the HE group was increased from 86.04% to 94.45%. However, the rate of mCHH was decreased 2.5-fold, from 10.70% to 4.24% (Fig. 1).

**Figure 2:**
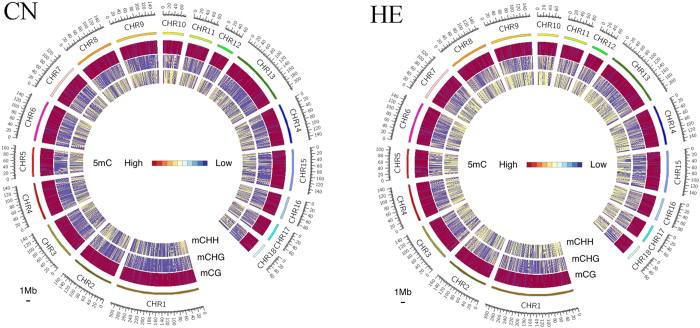
Density plot of 5-methylcytosine in various sequence contexts (mCG, mCHG, and mCHH). mC signifies 5-methylcytosine. H = A, C, or T. Chromosome numbers and scales are indicated on the periphery.

In the genome of each group, over 60% of the CpG sites were methylated, which is the primary DNA sequence context of cytosine methylation (Fig. 2). In the CN group, we detected abundant DNA methylation in CHH contexts. Although heat stress did not change the methylation level of the CpG sites in the genome, heat stress decreased the methylation level of the CHH contexts, which accounted for most of the difference between the groups.

### Sequence preferences analysis for methylation

**Figure 3:**
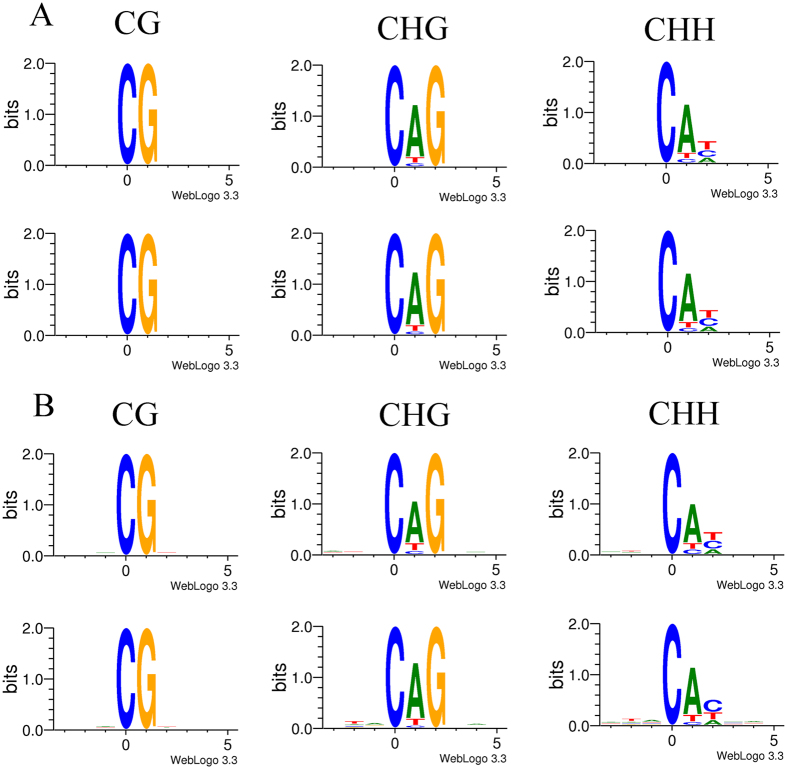
Sequence preferences for methylation in CG, CHG, and CHH contexts. Logos of sequence contexts that are preferentially methylated at the highest or lowest levels for 9-mer sequences in which the methylated cytosine is in the fourth position. (**A**) Regions of high methylation. (**B**) Regions of low methylation.

For CG context methylation, certain methylated C sites are defined as hypermethylation sites, at which the methylation level is over 75%; and some others are defined as sites of hypomethylation, at which the methylation level is less than 75%. For non-CG context methylation, hyper- and hypomethylation sites are defined as those at which the methylation levels are over or under 25%, respectively. In this study, we analyzed the relationship between sequence context and methylation preference. We calculated the percentage methylation of all possible 9-mer sequences in which the methylated cytosine was either in the fourth position (allowing an analysis of three nucleotides upstream of CG, CHG, and CHH methylation) or in the first position (allowing analysis of five nucleotides following the methylated cytosine; Fig. 3). In heavily methylated regions, there was no distinct difference between the two groups in the sequence enrichment based on the genomic regions. In contrast, however, in poorly methylated regions, a high level of sequence context preferences for methylation was observed, especially in non-CG contexts.

### DNA methylation levels of different functional regions

**Figure 4:**
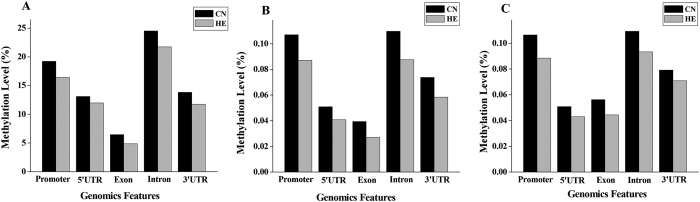
DNA methylation levels of different functional regions between the *Longissimus dorsi* muscles of the two groups. (**A**) CG regions. (**B**) CHG regions. (**C**) CHH regions. H = A, C, or T.

To study the global DNA methylation profile differences in the pig genome between control and constant heat stress conditions, we analyzed the DNA methylation levels of different genomic regions (Fig. 4). The comparison of average methylation levels showed that there were differential methylation levels in different components of the genome. A major proportion of methylated sites were present in the regions of promoters and introns. Among all the classes, the average methylation level of exons was the lowest. Furthermore, all five regions in the HE group exhibited lower methylation levels than did those in the CN group.

### DMR analysis

**Figure 5:**
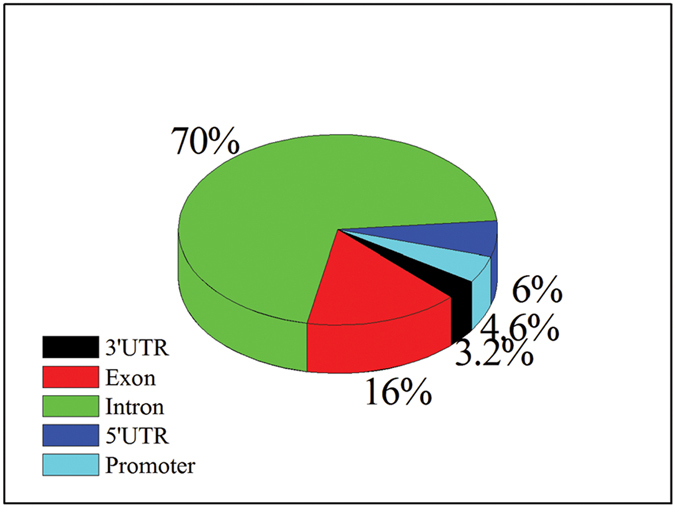
The distribution of DMR regions. DMR, differentially methylated region.

**Figure 6:**
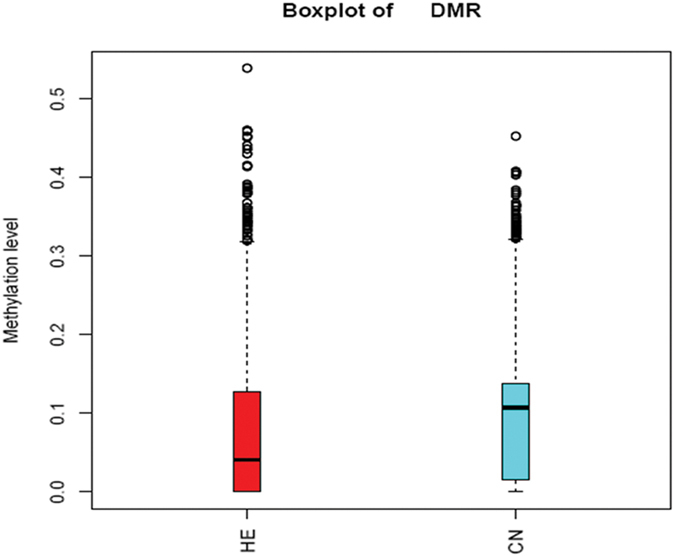
Methylation levels of DMRs in different groups. Boxes, quartiles 25–75%; black lines within boxes, median of the distribution (quartile 50%). DMR, differentially methylated region. HE, heat-exposed group. CN, control group.

To characterize the differences of genome methylation levels between samples under different environmental conditions, DMRs and differentially methylated genes (DMGs) were detected. A total of 57,147 DMRs were identified between the two groups, which corresponded to 1,422 DMGs (Table 3 and Supplementary Table). The methylated regions were mainly located in introns, which proportion exceeded 70% (Fig. 5). The results of a boxplot analysis of DMRs showed that the methylation level of the HE group was lower than that of the CN group (Fig. 6). Furthermore, we also found that some of these DMGs were involved in biological processes important for skeletal muscle development: energy metabolism (such as 6-phosphofructo-2-kinase/fructose-2,6-biphosphatase 1 [*PFKFB*], phosphoglycerate kinase 1 [*PGK1*], and pyruvate dehydrogenase kinase, isozyme 3 [*PDK3*]), transcription factors (such as cAMP responsive element binding [CREB], NF-κB- activating protein [*NKAP*], and signal transducer and activator of transcription 2 [*STAT2*]), Ca^2+^ homeostasis (chloride intracellular channel 2 [*CLIC2*], ryanodine receptor 3 [*RYR3*], and calcium channel, voltage-dependent, alpha 2/delta subunit 2 [*CACNA2D*]), protein kinases (such as calpain 2 [*CAPN2*] and protein kinase C [*PRKCA*]), and repair and stabilization of stressed proteins (such as αB-crystallin [*CRYAB*] *and* DnaJ [Hsp40] homolog, subfamily C, member 5 [*DNAJC5*]). However, gene ontogeny (GO) assignments and KEGG pathway analysis showed that these methylated genes were not significantly enriched in predicted process and signaling pathway (Supplementary Fig. 1 and Fig. 2).

### Association analysis between the DMGs and the differentially expressed genes (DEGs)

**Figure 7:**
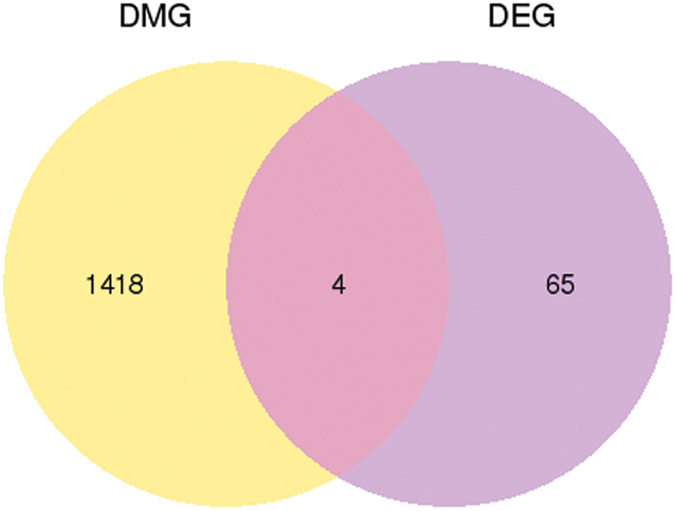
Numbers of genes that were differentially expressed in the two groups. DMG, differentially methylated gene; DEG, differentially expressed gene.

**Figure 8:**
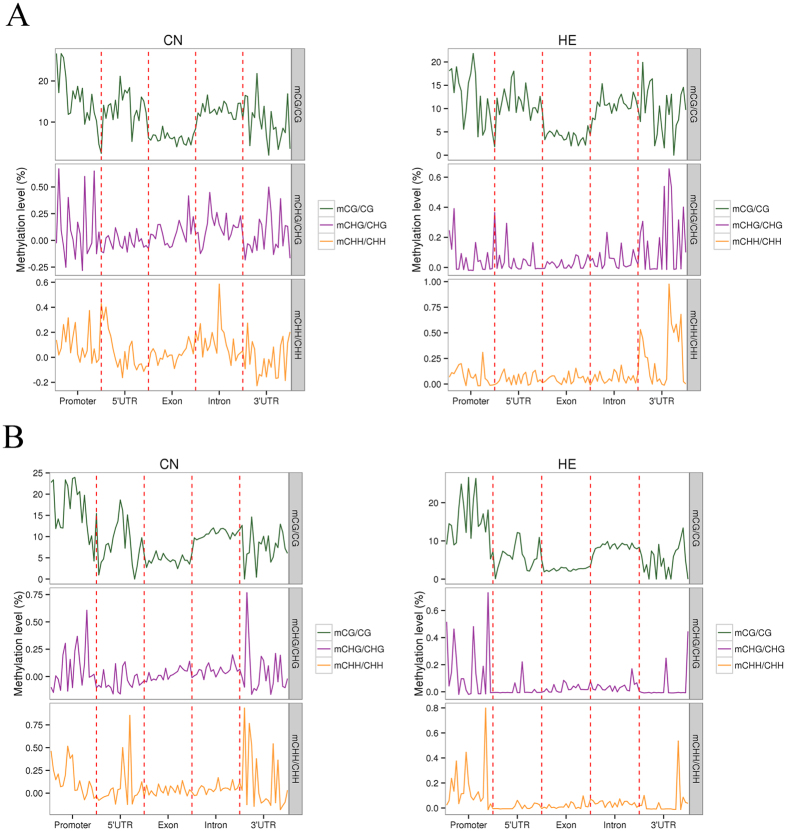
Association analysis of DMGs and DEGs. DEGs were divided into highly and lowly regulated types, according to their gene expression level. For each type of gene, methylation levels were analyzed in five functional element regions. (**A**) Highly regulated genes. (**B**) Lowly regulated genes. DEG, differentially expressed gene; DMG, differentially methylated gene. HE, heat-exposed group. CN, control group.

To explore the relationship between these DMGs and the DEGs found at the transcriptome level, an association analysis was performed. This identified four overlapped genes: *RYR3*, *PGK1*, *CRYAB*, and four and a half LIM domains 1 protein, isoform C *(FHL1C*; Fig. 7). In addition, DEGs were divided into highly regulated types and lowly regulated types, according to their gene expression level. For each type of gene, we analyzed the methylation level in five functional element regions (Fig. 8). For highly regulated genes, we observed a visible difference in methylation level between the CN and HE groups in the promoter regions for both CG and non-CG contexts. However, whereas a visible difference in methylation level was found in the exon regions of CHG contexts, differences in methylation levels were found in the 5′UTR, intron, and 3′UTR regions of CHH contexts. On the other hand, for lowly regulated genes, there was a visible difference in methylation level between CN and HE groups in the 5′UTR regions of CG context. A visible difference in methylation level was also found in the 3′UTR regions of CHG contexts, and a difference in methylation level was found in the promoter, 5′UTR, and 3′UTR regions of CHH contexts.

## Discussion

### DNA methylation profiles in skeletal muscle

Although global DNA methylation surveys have been performed on pigs^15^,^17^, this study is the first to systematically compare the genome-wide skeletal muscle methylation profiles between normal and heat-stressed pigs, which show different overall meat quality. Skeletal muscle is the major portion the animal used for human food, and its features have a direct impact on meat quality. Moreover, the DNA methylation status of promoter and gene body regions can affect gene expression via changes in chromatin structure or transcription efficiency^18^,^19^,^20^; therefore, we aimed to compare the genome-wide methylation patterns between heat-exposed and normal pigs to identify differentially methylated genes that may affect the quality of meat exposed to heat stress. From these analyses, we found that the intronic regions of the pig genome comprised a large proportion of DMRs (>70%) and that only a small proportion of DMRs were located in the 3′UTR, 5′UTR, and promoters. Furthermore, our previous study showed that the average daily gain (ADG) and average daily feed intake (ADFI) of heat-exposed pigs were both significantly decreased compared with those of control pigs^21^. These results could affect the observed changes in DNA methylation, in addition to direct heat stress. For decreased feed intake in heat-exposed pigs, food composition may be altered to compensate for decreased nutrients, which could offset a proportion of the observed methylation changes.

In mammals, most DNA methylation occurs at CpG sites, and asymmetric non-CpG methylation has been detected at appreciable levels in a few cell types^22^,^23^,^24^. Methylation in symmetrical sequences is preserved through cycles of DNA replication by maintenance DNA methyltransferases, which show a preference for hemimethylated substrates and methylate cytosines in the newly synthesized strand. Maintenance mechanisms for asymmetric methylation patterns are unknown, but must include de novo methylation after each cell division. Results of a recent study by Guo *et al.*^25^ showed that CpG methylation is always symmetrically methylated, whereas non-CpG sites are strand-specifically methylated in introns, short interspersed nuclear elements, and long interspersed nuclear elements^25^. They also showed that the skew of non-CpG methylation in introns is more pronounced at the boundaries and more significant for highly expressed genes. The results of this study provide further details regarding the biological significance of the various methylation patterns (CG, CHG, and CHH), particularly in the context of pigs.

### Key differentially methylated genes related to muscle development and meat quality

Porcine skeletal muscle development and growth have been shown to be associated with the differential expression of genes. In this study, several genes involved in muscle growth and development, including small muscle protein X-linked (*SMPX*), myosin, heavy chain 11 (*MYH11*), collagen, type XVI, alpha 1 (*COL16A1),* and collagen, type IV, alpha 3 (*COL4A3*), exhibited hypomethylation following constant heat stress. This result indicated that the expression of these genes might be upregulated. SMPX is upregulated in skeletal muscle by stretching and has been suggested to serve as both a transcription factor and a mechanosensor, possibly giving rise to changes in muscle fiber size and type. Thus, *SMPX* is thought to be a candidate gene for regulating muscle size. Schindeler *et al.*^26^ also found that SMPX might participate in the regulation of cytoskeletal dynamics through the Rac1/p38 pathway^26^. Additionally, differentiating C2C12 myoblasts overexpressing SMPX increase their susceptibility to fuse, forming large “myosacs” in an IGF-1 dependent manner^27^ further indicating a role for SMPX in the regulation of fiber size.

*COL16A1* is a gene suggested to have importance for cell adhesion^28^. COL4A3 was also identified as a structural component of collagen, which is known to affect meat tenderness. In turn, myosin is a major structural protein of the thick filament of the sarcomere^28^. A previous study demonstrated that MyHC isoform composition affects the quality of the meat originating from bovine muscles^29^ and is a major determinant of the phenotypic properties of muscles^30^. In this study, *MYH11* was identified as a differentially methylated gene between control and heat-stressed pigs. The results indicated that *MYH11* might be one of the major genes implicated in the differences in muscle fiber properties between control and heat-stressed pigs. Overall, the demonstration of upregulation of these genes suggests that constant heat stress might contribute to the development of disorders affecting muscle development.

### Key differentially methylated genes affecting muscle energy metabolism

In previous studies, many energy metabolism-related genes were found to be differentially expressed during porcine skeletal muscle growth and development^31^,^32^. In this study, the methylation levels of several genes encoding glycolytic enzymes, e.g., *PFKFB1*, *PGK1*, *and PDK3*, were found to be different between the two groups, which influenced the expression levels of these genes. PFKFB1, which encodes key enzymes involved in the glycolysis pathway, may exhibit upregulated expression in the HE group. This finding suggested that constant heat stress promoted muscle glucose metabolic processes. We assessed the methylation status of the 22048257–22049243 bp region of the *PDK3* gene, and found that relative hypomethylation of this region was associated with increased *PDK3* expression under constant heat stress. *PDK3* is one of four isozymes known to catalyze the phosphorylation of the α-subunit of the pyruvate dehydrogenase complex (PDC) in skeletal muscles^33^. PDC phosphorylation inhibits the conversion of pyruvate to acetyl-CoA and thus prevents the entry of glycolytic products into the mitochondria for oxidation, thereby disrupting the regulation of glucose homeostasis. The accumulation of lactic acid was found in heat-stressed pigs in our previous report^21^, which may be consistent with the increased *PDK3* level in skeletal muscles.

### Key differentially methylated genes affecting muscle lipid metabolism

Our previous transcriptome analysis results indicated that constant heat stress affected fat metabolism, such that the expression of skeletal muscle genes that encode the proteins involved in lipogenesis were lower in the HS group than in the CN group (unpublished data). In this study, we also identified some differentially methylated genes, e.g. carnitine palmitoyltransferase 1B (*CPT1B*), carnitine palmitoyltransferase 1A (*CPT1A*), and leptin receptor (*LEPR*), which were associated with muscle lipid metabolism. *CPT1B* is highly expressed in skeletal muscle, heart, and adipose tissues. *CPT1B* is the isoform of CPT1 that plays a crucial role in maintaining lipid homeostasis and therefore insulin sensitivity in skeletal muscle^34^. *LEPR* has also been reported to influence fat deposition^35^.

### Key differentially methylated genes associated with cellular defense and stress response

Heat-shock proteins 27 and 70 (*HSP27* and *HSP70*) and *CRYAB* have been identified as important HSPs involved in the repair and stabilization of stressed and damaged proteins^36^,^37^. In the unstressed state, HSPs are primarily found free and unbound in the cytosolic cell compartment. However, upon stress such as muscle damage, these proteins translocate, bind to, and accumulate in stressed and damaged structures^37^,^38^. HSPs have been shown to respond to environmental stress in the muscle^39^,^40^. A previous result showed that HSP27 is highly expressed in porcine skeletal muscles during heat stress^4^. Similarly, the methylation levels of some genes related to stress, such as *CRYAB* and *DNAJC5*, were found to be different between the groups in this study. As a member of the *HSP40* family, *DNAJC5* is mainly involved in the regulation of HSP70 ATPase activity. HSP70s and their associated cochaperones participate in numerous processes essential to cell survival under both normal and stressed conditions^41^. They assist, for example, in protein folding and translocation across membranes, assembly and disassembly of protein complexes, presentation of substrates for degradation, and suppression of protein aggregation^42^.

### Key differentially methylated genes associated with calcium signaling pathways

Activated and disrupted regulation of the intracellular Ca^2+^ concentration is responsible for the increased metabolism of meat^43^. In addition, the elevation of corticosteroids is associated with a decline in the Ca^2+^ concentration within the endoplasmic reticulum lumen, which contributes to the imbalance of total cellular Ca^2+ ^44. In particular, a previous study found a causative mutation in the ryanodine receptor gene (*RyR*), which is closely linked to porcine stress syndrome in pigs and usually accompanied by poor meat quality^45^. More recent work has demonstrated that CLIC2 modulates intracellular Ca^2+^ homeostasis through RYRs, suggesting that CLIC2 may alter the calcium homeostasis of any tissue through RYRs^46^. In this study, both the *CLIC2* and *RYR* genes were identified as differentially methylated genes between the groups, which suggested that constant heat stress affects calcium signaling pathways in the skeletal muscles.

### Key differentially methylated genes associated with transcription factors

CREB protein, a stimulus-induced transcription factor, activates the transcription of target genes in response to a diverse array of stimuli, including peptide hormones, growth factors, and neuronal activity^47^. A reduction in methylation during heat conditioning was observed at a CREB site in a previous study^48^. Our results also identified the *CREB* gene as a differentially methylated gene, indicating that constant heat stress induced the activation of some transcription factors. Furthermore, compared with the CN group, we found increased DNA methylation of *NKAP* in the HE group. *NKAP* encodes the NF-κB-activating protein, which is a nuclear protein involved in the activation of NF-κB transcription^49^. NF-κB is known to positively regulate the transcription of several inflammatory cytokines, such as IL-1β, interferon-γ, and TNF-α^50^, which have been shown to decrease glucose-stimulated insulin secretion^51^,^52^,^53^. Modulation of NF-κB could provide a possible mechanism for increased insulin secretion in NKAP-silenced cells and in islets that exhibit higher methylation and lower expression of *NKAP*^54^. This result may therefore imply that constant heat stress could suppress the activation of the NF-κB signaling pathway in skeletal muscle.

In the current study, we used DNA methylation profiling to elucidate possible regulatory mechanisms of heat stress on pig skeletal muscle development and meat quality. Accordingly, additional studies are required to evaluate the expression and epigenetic effects of the genes identified in this study, as well as epigenetic reprogramming and tissue development in heat-stressed pigs.

## Conclusions

This study provided a comprehensive analysis of DNA methylation profiles of pig skeletal muscle between normal and constant heat stress conditions. We identified DMRs and genes associated with these regions. Pathway and network analysis of these differentially methylated genes revealed a number of candidate genes that might affect muscle development and meat quality. The results of this study might therefore provide new clues for deciphering the epigenetic mechanisms of pig skeletal muscle growth and development and will likely contribute to the improvement of meat quality.

## Methods

### Ethics statement

The animal component of this study was conducted in accordance with the Guidelines for Experimental Animals, established by the Ministry of Science and Technology (Beijing, China). The animal experiments were approved by the Science Research Department of the Institute of Animal Sciences, Chinese Academy of Agricultural Sciences (CAAS; Beijing, China). The researchers who performed the animal experiments possessed qualified certifications issued by the Beijing Association for Laboratory Animal Care (Beijing, China).

### Animals and experimental design

A total of 16 castrated male DLY pigs (crossbreeds between Landrace ×Yorkshire sows and Duroc boars) were selected from eight litters (body weight: 79.0 ± 1.5 kg) at a pig breeding farm in Beijing, China. To eliminate differences in genetic background, two pigs from each litter were allocated to one of two treatments: (1) control treatment without heat stress (22 °C; CON, n = 8) and (2) constant heat stress (30 °C; H30, n = 8). All pigs were individually caged in the environmental control cabin of the State Key Laboratory of Animal Nutrition and had ad libitum access to water and feed. The animals were housed under a 14-h light/10-h dark cycle; the environmental control cabin had a relative humidity of 55% ± 5%.

Prior to the experiment, the animals were given 7 days to acclimatize to the 22 °C environmental control cabin. After a 21-day experimental period, the pigs were slaughtered via electrical stunning using a head-only electric stun tong apparatus (Xingye Butchery Machinery Co. Ltd., Changde, China), followed by exsanguination. *Longissimus dorsi* muscles from the tenth thoracic vertebra of the right side of each carcass were collected and snap-frozen in liquid nitrogen immediately in order to ensure high RNA quality and were then stored at −80 °C until use.

### DNA extraction and preparation

Genomic DNA was extracted using a DNeasy Blood & Tissue Kit (Qiagen GmbH, Hilden, Germany), according to the manufacturer’s recommendations. Frozen tissues were used directly. DNA quality was monitored using 1% agarose gel electrophoresis. DNA purity was assessed via a NanoPhotometer spectrophotometer (Implen USA, Inc., Westlake Village, CA, USA). DNA concentrations were measured using a Qubit DNA Assay Kit with a Qubit 2.0 Fluorometer (Life Technologies, Carlsbad, CA, USA).

### Library preparation and quantification

A total of 5.2 μg of genomic DNA spiked with 26 ng lambda DNA was fragmented by sonication to 200–300 bp with the Covaris S220 Focused-ultra sonicator (Covaris, Woburn, MA, USA) followed by end repair and adenylation. Cytosine-methylated barcodes were ligated to sonicated DNA as per the manufacturer’s instructions. The barcoded DNA fragments were then treated twice with bisulfite using an EZ DNA Methylation-GoldTM Kit (Zymo Research, Irvine, CA, USA). The resulting single-stranded DNA fragments were amplified by polymerase chain reaction (PCR) using the KAPA HiFi HotStart Uracil + ReadyMix (2X) (Kapa Biosystems, Wilmington, MA, USA).

Library concentrations were quantified using a Qubit 2.0 Fluorometer and quantitative PCR, and the insert size was checked on an Agilent Bioanalyzer 2100 system (Agilent Technologies, Santa Clara, CA, USA).

### Data analysis

#### Quality control

Read sequences produced by the Illumina pipeline in FastQ format were first preprocessed through in-house Perl scripts. First, because a subset of reads contained all of a portion of the 3′ adapter oligonucleotide sequence, every read was scanned for the adapter sequence, and if detected the read was filtered out. Second, since some reads contained one or more Ns (unknown bases) in their sequences, the percentage of Ns in each read was calculated, and if the percentage of Ns was larger than 10%, the read was removed. Third, reads with low quality (PHRED score ≤5, and percentage of the low quality bases ≥50%) were trimmed. At the same time, the Q20, Q30, and GC content of the data were calculated. The remaining reads that passed the filters were called as clean reads and all of the subsequent analyses were based on these.

### Read mapping to the reference genome

Bismark software (version 0.12.5)^55^ was used to perform alignments of bisulfite-treated reads to a reference genome using the default parameters. The reference genome was first transformed into a bisulfite-converted version (C-to-T and G-to-A conversions) and then indexed using bowtie2^56^. Sequence reads were also transformed into fully bisulfite-converted versions (C-to-T and G-to-A conversions) before they were aligned to similarly converted versions of the genome in a directional manner. Sequence reads that produced a unique best alignment from the two alignment processes (original top and bottom strand) were then compared to the normal genomic sequence, and the methylation state of all cytosine positions in the read was inferred. The same reads that aligned to the same regions of the genome were regarded as duplicated reads. The sequencing depth and coverage were summarized using deduplicated reads. The sodium bisulfite non-conversion rate was calculated as the percentage of cytosines sequenced at cytosine reference positions in the lambda genome.

### Estimating methylation levels

To identify the sites of methylation, we modeled the sum 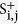 of methylated counts as a binomial (Bin) random variable with a methylation rate as follows:


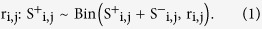


We employed a sliding-window approach, which is conceptually similar to approaches that have been used for bulk bisulfite sequencing (http://www.bioconductor.org/packages/2.13/bioc/html/bsseq.html). Using a window size w = 3,000 bp and step size of 600 bp^57^, the sum of methylated and unmethylated read counts in each window were calculated. The methylation level (ML) for each C site showed the fraction of methylated Cs and was defined as:


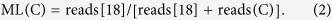


The calculated ML value was further corrected using the bisulfite non-conversion rate according to previous studies^58^. Given the bisulfite non-conversion rate r, the corrected ML was estimated as:


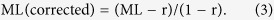


### Differentially methylated region analysis

Differentially methylated regions (DMRs) were identified using swDMR software (http://122.228.158.106/swDMR/), using a sliding-window approach. The window was set to 1000 bp and the step length was 100 bp. A Fisher test was then implemented to detect the DMRs. We defined regions as DMRs that had corrected *P*-value of less than 0.05, reads coverage of greater than 5, fold-change values of greater than 2 and false discovery rate of less than 0.05.

### GO and KEGG enrichment analysis of DMR-related genes

GO enrichment analysis of genes related to DMRs was implemented by the GOseq R package^59^, in which gene length bias was corrected. KOBAS software^60^ was used to test the statistical enrichment of DMR-related genes in KEGG pathways. GO terms and KEGG with corrected *P*-values of less than 0.05 were considered significantly enriched by DMR-related genes.

### Transcriptome analysis

#### Sample preparation for RNA-seq analysis

mRNA was purified from total RNA using poly-T oligo-attached magnetic beads. Fragmentation was performed using divalent cations under elevated temperatures in a proprietary Illumina fragmentation buffer. First-strand cDNA was synthesized using random oligonucleotides and SuperScript II (Life Technologies Inc, Rockville, MD, USA). Second-strand cDNA synthesis was then performed using DNA polymerase I and RNase H. Remaining overhangs were converted into blunt ends using exonuclease and polymerase. After the adenylation of the 3′ ends of the DNA fragments, Illumina PE adapter oligonucleotides were ligated in preparation for hybridization. To preferentially select cDNA fragments 200 bp in length, the library fragments were purified using the AMPure XP system (Beckman Coulter, Beverly, MA, USA). DNA fragments with adaptors ligated on both ends were selectively enriched using the Illumina PCR Primer Cocktail with a 10-cycle PCR protocol. The products were then purified (AMPure XP system) and quantified using Agilent high-sensitivity DNA assays on the Agilent Bioanalyzer 2100 system.

### Clustering and sequencing

Clustering of the index-coded samples was performed on a cBot Cluster Generation System using the TruSeq PE Cluster Kit v3-cBot-HS (Illumina). After cluster generation, the library preparations were sequenced on an Illumina Hiseq 2000 platform and 100-bp paired-end reads were generated (Novogene, Beijing, China).

### Quantification of gene expression levels

After quality control and filtering of raw data, clean reads were aligned to the *S. scrofa* 10.2 reference genome using TopHat v1.4.0^61^, which was chosen as it could generate a database of splice junctions based on the gene model annotation file to provide better mapping results.

HTSeq v0.5.3 was used to count the read numbers mapped to each gene. The reads per kilobase of exon model per million mapped reads (RPKM) of each gene was calculated based on the length of the gene and the number of reads mapped to this gene.

### Differential gene expression analysis

Differential gene expression between the control and heat stress groups was analyzed using the DESeq R package (1.8.3). The *P*-values were adjusted using the Benjamini & Hochberg method^62^. A corrected *P*-value of 0.05 was set as the threshold for significantly different expression.

### Association analysis

We estimated the associations between the DMGs and the differentially expressed genes (DEGs) by Pearson correlation. In addition, DEGs were divided into highly regulated types and lowly regulated types, according to the results of transcriptome analysis. For each type of gene, we analyzed the methylation level in five functional element regions. Each gene function element was divided into 20 bins according to the location. The methylation level of each bin was calculated sequentially, and the average methylation levels in bin1, bin2, bin3 … bin20 of all genes were then calculated.

## Additional Information

**How to cite this article**: Hao, Y. *et al.* Genome-wide DNA methylation profiles changes associated with constant heat stress in pigs as measured by bisulfite sequencing. *Sci. Rep.* **6**, 27507; doi: 10.1038/srep27507 (2016).

## Supplementary Material

Supplementary Information

## Figures and Tables

**Table 1:** Data generated by genome-wide bisulfite sequencing.

Samples	Raw reads	Raw bases (G)	Clean reads	Clean bases (G)	Total mapped reads	Mapping rate (%)	Duplication rate (%)
CN	680456464	85.06	246647114	61.66	152148958	61.69	4.52
HE	625512604	78.18	250285737	62.58	164704774	65.81	17.81

HE, heat-exposed group. CN, control group.

**Table 2:** Genome-wide methylation levels of the two groups.

Samples	mC percent (%)	mCpG percent (%)	mCHG percent (%)	mCHH percent (%)
CN	3.95	67.62	0.59	0.58
HE	3.40	67.54	0.21	0.20

HE, heat-exposed group. CN, control group.

**Table 3:** Differentially methylated genes shared by HE and CN groups.

No.	Gene ID	Gene symbol	Gene description	Up/down
1	100144303	ACE2	angiotensin I converting enzyme (peptidyl-dipeptidase A) 2	↑(1)↓(2)
2	448980	ACSL4	acyl-CoA synthetase long-chain family member 4	↑(3)↓(2)
3	100517353	ADD1	adducin 1 (alpha)	↓
4	100524062	AIFM1	apoptosis-inducing factor, mitochondrion-associated, 1	↓(2)
5	397245	ATP1B4	ATPase, Na + /K + transporting, beta 4 polypeptide	↑
6	100514935	ATP6AP2	ATPase, H + transporting, lysosomal accessory protein 2	↓
7	397159	ATP7A	ATPase, Cu + + transporting, alpha polypeptide	↓
8	100517557	CACNA2D2	calcium channel, voltage-dependent, alpha 2/delta subunit 2	↓
9	396585	CACNB4	calcium channel, voltage-dependent, beta 4 subunit	↑
10	397393	CAPN2	calpain 2, (m/II) large subunit	↑
11	100522971	CAPN6	calpain 6	↓
12	100152881	CASK	calcium/calmodulin-dependent serine protein kinase (MAGUK family)	↑(4) ↓(3)
13	100516055	CLIC2	chloride intracellular channel 2	↑
14	397502	COL4A3	collagen, type IV, alpha 3 (Goodpasture antigen)	↓
15	100519180	COL4A5	collagen, type IV, alpha 5	↑(3)↓(3)
16	100737666	COL16A1	collagen, type XVI, alpha 1	↓
17	399527	CPT1A	carnitine palmitoyltransferase 1A (liver)	↑
18	399528	CPT1B	carnitine palmitoyltransferase 1B (muscle)	↑
19	100142688	CREB3L4	cAMP responsive element binding protein 3-like 4	↓
20	100519789	CRYAB	crystallin, alpha B	↓
21	403331	CYP19A1	cytochrome P450 19A1	↓
22	100626784	DMAP1	DNA methyltransferase 1 associated protein 1	↓
23	100621635	DNAJC5	DnaJ (Hsp40) homolog, subfamily C, member 5	↑(1)↓(1)
24	397083	EGF	epidermal growth factor	↓
25	397070	EGFR	epidermal growth factor receptor	↓
26	397667	FHL1C	four and a half LIM domains 1 protein, isoform C	↓
27	397108	GP91-PHOX	NADPH oxidase heavy chain subunit	↑
28	100522160	HOXB3	homeobox B3	↓
29	100524920	HTR2C	5-hydroxytryptamine (serotonin) receptor 2C, G protein-coupled	↓
30	733692	IL15RA	interleukin 15 receptor, alpha	↓
31	396801	LDLR	low density lipoprotein receptor	↑
32	396836	LEPR	leptin receptor	↓
33	100510925	LNX2	ligand of numb-protein X 2	↑
34	100622469	METTL1	methyltransferase like 1	↑
35	100621533	MYH11	myosin, heavy chain 11, smooth muscle	↓
36	100524288	MYLK2	myosin light chain kinase 2	↑
37	100514505	NKAP	NFKB activating protein	↑
38	100739747	NOX1	NADPH oxidase 1	↑
39	100294678	PDHA1	pyruvate dehydrogenase (lipoamide) alpha 1	↓
40	100153858	PDK3	pyruvate dehydrogenase kinase, isozyme 3	↑(1)↓(1)
41	100233197	PFKFB1	6-phosphofructo-2-kinase/fructose-2,6-biphosphatase 1	↓
42	407608	PGK1	phosphoglycerate kinase 1	↑
43	397013	PPARGC-1	peroxisome proliferator activated receptor gamma, coactivator 1 alpha	↑(1)↓(1)
44	397184	PRKCA	protein kinase C, alpha	↓
45	100627149	RAC1	ras-related C3 botulinum toxin substrate 1 (rho family, small GTP binding protein Rac1)	↑
46	396888	RYR3	ryanodine receptor 3	↓
47	780438	SMPX	small muscle protein, X-linked	↓
48	100623576	SNW1	SNW domain containing 1	↑
49	396923	STAT2	signal transducer and activator of transcription 2, 113 kDa	↓
50	100517796	TAB3	TGF-beta activated kinase 1/MAP3K7 binding protein 3	↓(2)
51	100519300	TGFBI	transforming growth factor, beta-induced, 68 kDa	↓
52	397384	TLR8	toll-like receptor 8	↓
53	414906	TNNT3	troponin T type 3 (skeletal, fast)	↑
54	100523109	ZDHHC9	zinc finger, DHHC-type containing 9	↓

HE, heat-exposed group. CN, control group.
