# Supplementary material for: Genome-wide DNA methylation profiles changes associated with constant heat stress in pigs as measured by bisulfite sequencing
Source: Sci Rep. 2016 Jun 6;6:27507. doi: 10.1038/srep27507 (PMC4893741; doi:10.1038/srep27507)
Supplement: Supplementary Information [file srep27507-s1.pdf]

Genome-wide DNA methylation profiles changes associated with constant heat stress in pigs as measured by bisulfite sequencing

Yue Hao, Yanjun Cui, Xianhong Gu*

State Key Laboratory of Animal Nutrition, Institute of Animal Sciences, Chinese Academy of Agricultural Sciences, Beijing, People's Republic of China

*Corresponding author

E-mail: guxianhong@vip.sina.com

Supplemental files

Supplemental file 1. Gene ontology (GO) functional annotations for the differentially methylated genes. Bar graphs showed three independent GO) = categories: cellular components, molecular functions, and biological processes. In each bar graph, the abscissa represents the number of differentially expressed genes, and the ordinate is the GO terms.

Supplemental file 2. KEGG enrichment pathway terms for the differentially methylated genes. The ordinate represent the enriched pathway terms, and the abscissa represent the richness factor of these terms; the size of the spots represent the number of differently expressed genes enriched in each pathway, while the color of the spot represents the *Q*-value of each pathway.

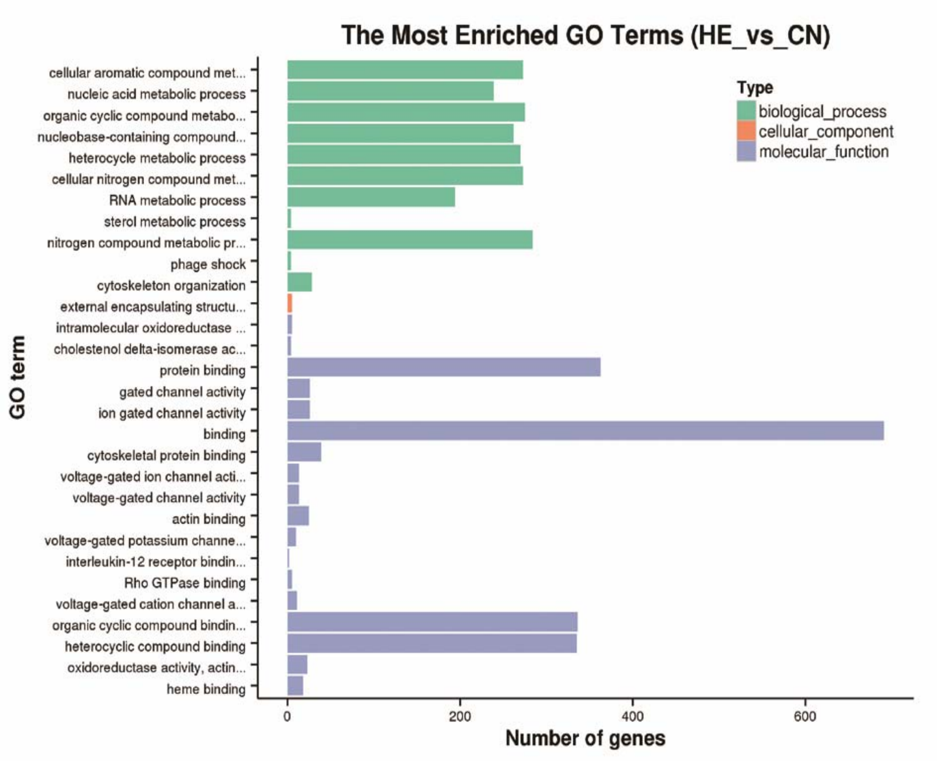
The Most Enriched GO Terms (HE_vs_CN)
GO term
cellular aromatic compound met...
nucleic acid metabolic process
organic cyclic compound metabo...
nucleobase-containing compound...
heterocycle metabolic process
cellular nitrogen compound met...
RNA metabolic process
sterol metabolic process
nitrogen compound metabolic pr...
phage shock
cytoskeleton organization
external encapsulating structu...
intramolecular oxidoreductase ...
cholestenol delta-isomerase ac...
protein binding
gated channel activity
ion gated channel activity
binding
cytoskeletal protein binding
voltage-gated ion channel acti...
voltage-gated channel activity
actin binding
voltage-gated potassium channe...
interleukin-12 receptor bindin...
Rho GTPase binding
voltage-gated cation channel a...
organic cyclic compound bindin...
heterocyclic compound binding
oxidoreductase activity, actin...
heme binding
0
200
400
600
Number of genes
Type
biological_process
cellular_component
molecular_function

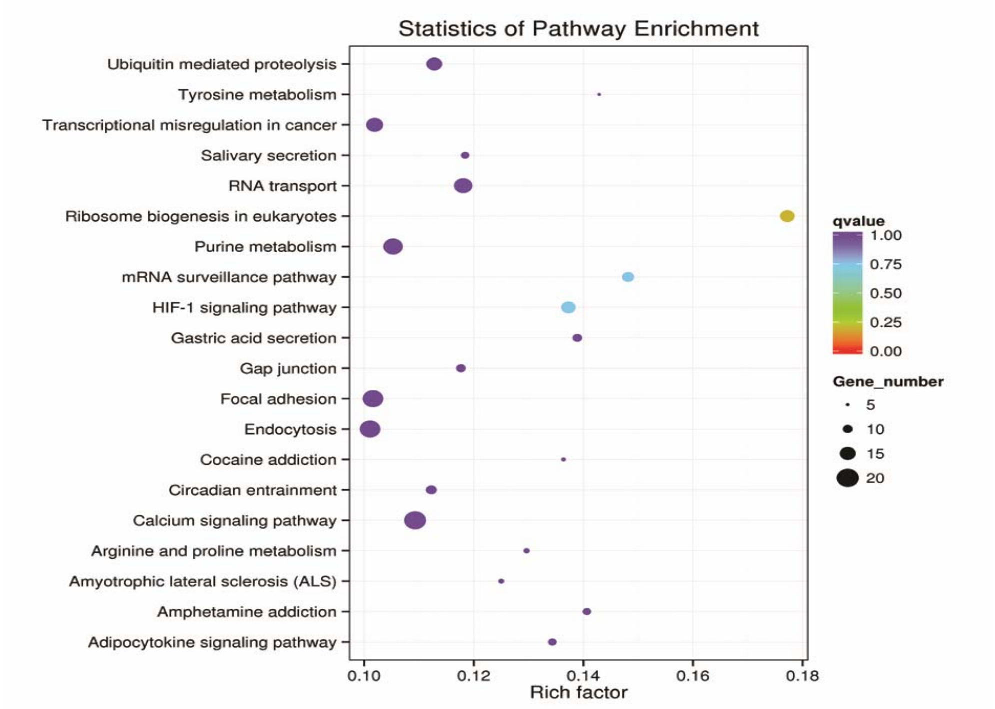
Statistics of Pathway Enrichment
Ubiquitin mediated proteolysis
Tyrosine metabolism
Transcriptional misregulation in cancer
Salivary secretion
RNA transport
Ribosome biogenesis in eukaryotes
Purine metabolism
mRNA surveillance pathway
HIF-1 signaling pathway
Gastric acid secretion
Gap junction
Focal adhesion
Endocytosis
Cocaine addiction
Circadian entrainment
Calcium signaling pathway
Arginine and proline metabolism
Amyotrophic lateral sclerosis (ALS)
Amphetamine addiction
Adipocytokine signaling pathway
0.10
0.12
0.14
0.16
0.18
Rich factor
qvalue
1.00
0.75
0.50
0.25
0.00
Gene_number
5
10
15
20
